# How to target the coronoid from the dorsal cortex

**DOI:** 10.1016/j.jseint.2025.08.008

**Published:** 2025-09-09

**Authors:** Simone Cassin, Valeria Vismara, Aurelien Traverso, Christos Koukos, Pietro Simone Randelli, Paolo Arrigoni

**Affiliations:** a Scuola Di Specializzazione in Ortopedia e Traumatologia, Università Degli Studi Di Milano, Milan, Italy; b Department of Orthopaedics and Traumatology, Centre Hopitalier Universitaire Vaudois (CHUV), Lausanne, Switzerland; c Sports Trauma & Pain Institute, Thessaloniki, Greece; d Clinica Ortopedica, Azienda Socio Sanitaria Territoriale Centro Specialistico Ortopedico Traumatologico Gaetano Pini-CTO, Milan, Italy; e Laboratory of Applied Biomechanics, Department of Biomedical Sciences for Health, Università Degli Studi Di Milano, Milan, Italy; f Research Center for Adult and Pediatric Rheumatic Diseases (RECAP-RD), Department of Biomedical Sciences for Health, Università Degli Studi Di Milano, Milan, Italy

**Keywords:** Coronoid process, Dorsal cortex, CT Scan, Fracture fixation, Range, Surgical practice

## Abstract

**Background:**

The coronoid process of the ulna is crucial for both anteroposterior and axial elbow stability. Currently, there is no safe and X-ray–free method for targeting the coronoid from the posterior cortex of the ulna, for temporary or permanent fixation. This study aims to define a range, easy to use in surgical practice, to safely target the coronoid process from the dorsal cortex of the ulna and to normalize the range based on the inter-epicondylar distance (IED).

**Methods:**

For the study, 3 different parameters were assessed: the APEX (olecranon to coronoid apex distance), BASE (olecranon to coronoid base distance), and the IED from 109 computed tomographies.

**Results:**

The mean APEX was 20.6 mm (19.3 mm in females and 21.3 mm in males), and the mean BASE was 33.9 mm (31.9 mm in females and 35.1 mm in males). The mean IED was 59 mm (53.3 mm in females and 62.3 mm in males), the mean ratio between APEX and IED was 0.3 (0.4 in females and 0.3 in males), the mean ratio between BASE and IED was 0.6 (0.6 in males and females). These data were significantly different in males and females (*P* < .05).

**Conclusions:**

This study contributes to establishing a practical range for the safe targeting of the coronoid process from the dorsal cortex of the ulna in surgical practice. A safe approach to the coronoid can be achieved by maintaining a perpendicular trajectory to the dorsal cortex of the olecranon within a safe range of 2 to 3.5 cm from its most prominent point.

The coronoid process of the ulna is crucial for both anteroposterior and axial elbow stability, being a bony buttress to posterior ulnar displacement and giving the insertion to the anterior band of the medial collateral ligament, the brachialis and the anterior capsule at its base.^18^

Coronoid process of the ulna fractures can be classified according to their height, as described by Regan and Morrey,^17^ or to their site, size and injury mechanism, as described by O′ Driscoll.^16^ Significant elbow instability has been shown to occur when 50% or more of the coronoid process is missing (grade 2 of Morrey's classification).^6^^,^^10^ Therefore, it is crucial to fix it to get a stable elbow. Several methods of fixation have been described: suture,^2^^,^^8^ Kirschner wires,^5^^,^^9^ lag screws^4^^,^^14^ and buttress plates^4^^,^^18^ are the main ones.

Classically, open approaches were used, but more recently, arthroscopy-assisted fixation has increasingly risen.^3^ Despite the surgical approach, during the surgical procedure it is common to temporarily or permanently fix the coronoid using K-wires inserted from the dorsal cortex of the ulna. Fluoroscopic guidance is typically used, and to date there is no safe X-ray–free method for targeting the coronoid from the dorsal cortex.

The primary aim of this study is to define a range, easy to use in surgical practice, to safely target the coronoid process from the dorsal cortex of the ulna, helping orthopedic surgeons in its fracture fixation. The secondary aim of this study is to normalize the range based on the inter-epicondylar distance (IED) in order to express it inde-pendently of the patient's general characteristics.

## Material and Methods

We analyzed retrospectively all elbow computed tomographies (CTs) performed at 90° degrees of flexion from January 2019 to December 2022 at our institution in patients undergoing an acute trauma injury. We excluded all the examinations with bony injuries, major ligamentous damages, previous surgeries, and arthritic degeneration. All CT scans consisted of axial images with a 1.25 mm slice thickness, which were then reconstructed in the axial, coronal, and sagittal planes. All measurements were taken on the slices where the coronoid process of the ulna and the IED were most prominent.

This study was conducted in accordance with the Declaration of Helsinki (1964) and its subsequent amendments, as well as the ethical standards of the institutional and/or national research committee. Informed written consent was obtained from all patients after thorough explanation and approval was granted by our institutional ethics committee (CT-Elbow Fractures, No. 139-2021).

**Figure 1:**
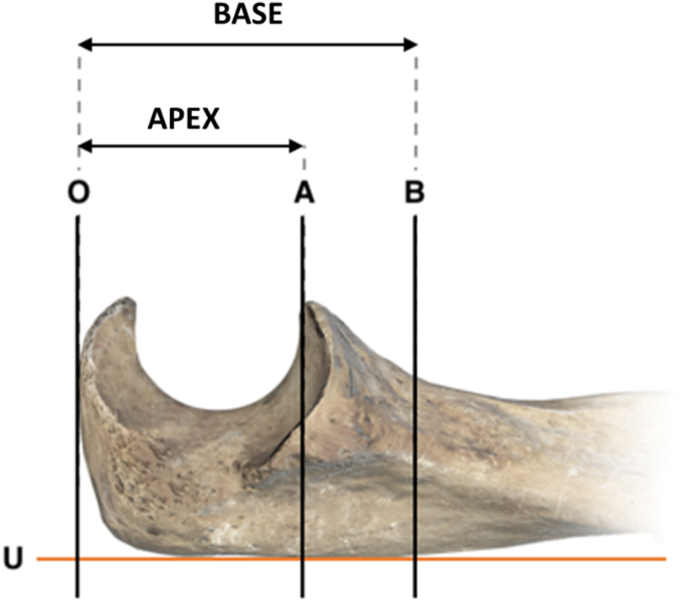
Illustration of the measurement technique. The distances between the most prominent point of the olecranon dorsal cortex and the coronoid APEX and the coronoid BASE were measured on sagittal cuts. Four lines were identified: line U (tangent to the ulnar dorsal cortex), line O (perpendicular to U, tangent to the olecranon posterior cortex), line A (perpendicular to U, passing through the coronoid's APEX), line B (perpendicular to U, passing through the coronoid's BASE). With these references, 2 distances were calculated, APEX (olecranon to coronoid apex distance) and BASE (olecranon to coronoid base distance).

All the CTs were reviewed by mutual agreement by 2 independent examinators. The IED was measured on coronal cuts using AGFA XERO Universal Viewer^1^ (AGFA Healthcare, Mortsel, Belgium). The distances between the most prominent point of the olecranon dorsal cortex and the coronoid APEX and the coronoid BASE were measured on sagittal cuts (Fig. 1). We identified 4 lines.

- line U: tangent to the ulnar dorsal cortex;
- line O: perpendicular to U, tangent to the olecranon posterior cortex;
- line A: perpendicular to U, passing through the coronoid's APEX;
- line B: perpendicular to U, passing through the coronoid's BASE.

Given these references, we measured 2 distances, in mm.

- olecranon to coronoid apex distance (APEX): distance from O to A;
- olecranon to coronoid base distance (BASE): distance from O to B.

In addition, we normalized the value assessed with the IED.

A statistical analysis was finally performed using Jamovi 2.5^11^ (Jamovi Project, Sydney, Australia). The Shapiro–Wilk test was used to evaluate the normality of data distributions. The Levene test was used to evaluate the homoscedasticity of data distributions. Differences between groups were evaluated with unpaired Student's t-test, Welch's t-test, or Mann–Whitney *U* test, according to the characteristics of data distributions.

## Results

Overall, 109 CTs performed between January 2019 and December 2022, were retrospectively analyzed. Of these, 69 were males and 40 were females. The mean APEX was 20.6 mm (interquartile [IQ]: 18.4 mm-22.7 mm; mean standard deviation [MSD]: 3.3), and the mean BASE was 33.9 mm (IQ: 31.4 mm-36.8 mm; MSD: 4.6). The mean IED was 59 mm (IQ: 55.0 mm-63.2 mm; MSD: 5.8), the mean ratio between APEX and IED was 0.3 (IQ: 0.3-0.4), the mean ratio between BASE and IED was 0.6 (IQ: 0.5-0.6) (Table I).

**Table I:** Descriptive statistical analysis of the overall measurements expressed in mm (APEX, BASE, and IED).

	APEX	BASE	IED	APEX/IED	BASE/IED
Mean	20.6	33.9	59.0	0.3	0.6
Mean Square Deviation	3.3	4.6	5.8	0.1	0.1
25^th^ Percentile	18.4	31.4	55.0	0.3	0.5
50^th^ Percentile	20.8	34.0	59.3	0.3	0.6
75^th^ Percentile	22.7	36.8	63.2	0.4	0.6

*IED*, interepicondylar distance; *APEX*, olecranon to coronoid apex distance; *BASE*, olecranon to coronoid base distance; *APEX/IED*, olecranon to coronoid apex distance/interepicondylar distance; *BASE/IED*, olecranon to coronoid base distance/interepicondylar distance.

APEX/IED and BASE/IED ratios are absolute values.

The mean APEX was 19.3 mm in females and 21.3 mm in males; the mean BASE was 31.9 mm in female, 35.1 mm in males; the mean IED was 53.3 mm in females, 62.3 mm in males; the mean ratio between APEX and IED was 0.4 in females, 0.3 in males; and the mean ratio between BASE and IED was 0.6 in males and females (Table II).

**Table II:** Descriptive and inferential statistical analysis of the measurements divided by sex.

	SEX	APEX	BASE	IED	APEX/IED	BASE/IED
N	F	40	40	40	40	40
	M	69	69	69	69	69
Mean	F	19.3	31.9	53.3	0.4	0.6
	M	21.3	35.1	62.3	0.3	0.6
Mean Square Deviation	F	3.0	3.6	3.3	0.1	0.1
	M	3.3	4.7	4.0	0.1	0.1
25^th^ Percentile	F	17.2	29.3	51.0	0.3	0.6
	M	19.7	32.9	60.0	0.3	0.5
50^th^ Percentile	F	19.1	32.2	53.5	0.4	0.6
	M	21.3	35.0	62.7	0.3	0.6
75^th^ Percentile	F	21.4	33.8	55.5	0.4	0.6
	M	23.5	38.0	64.4	0.4	0.6
*P* (Student's *t*-test)	N/A	.003	<.001	<.001	.042	.010

*APEX*, olecranon to coronoid apex distance; *BASE*, olecranon to coronoid base distance; *APEX/IED*, olecranon to coronoid apex distance/interepicondylar distance; *BASE/IED*, olecranon to coronoid base distance/inter-epicondylar distance.

APEX, BASE, and IED are expressed in mm, while the ratios are absolute values. According to Student's *t*-test, there is a statistically significant difference of APEX, BASE, IED and APEX and BASE/IED ratio between sex.

According to Student's t-test, APEX, BASE and the ratios between APEX, BASE and IED significantly differ in males and females (*P* < .05) (Table II).

**Figure 2:**
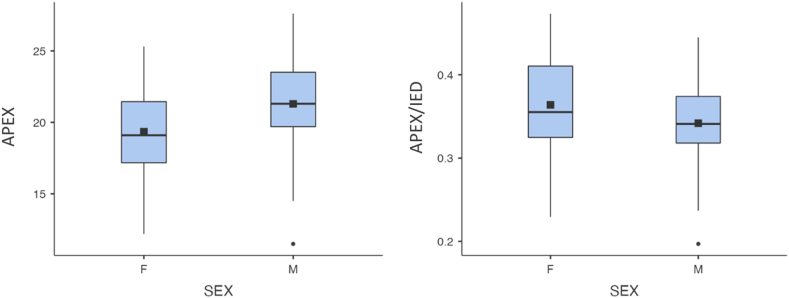
Box plot graph of the measurements of APEX and APEX/IED ratio, divided by sex. *APEX*, olecranon to coronoid apex distance; *APEX/IED*, olecranon to coronoid apex distance/interepicondylar distance.

**Figure 3:**
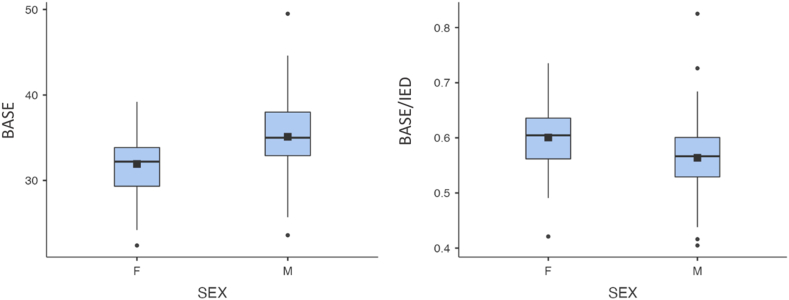
Box plot graph of the measurements of base and BASE/IED ratio, divided by sex. BASE, olecranon to coronoid base distance; BASE/IED, olecranon to coronoid base distance/interepicondylar distance.

All results are shown fully in the following table and graphs (Fig. 2, Fig. 3).

Based on these considerations, the quantity of CT scans analyzed is sufficient to conduct our analyses with a reliable degree of precision.

## Discussion

Although uncommon,^15^ fractures of the coronoid process of the ulna can significantly compromise elbow stability, often requiring internal reduction and fixation to restore it.

The ability to precisely target the APEX and the BASE of the coronoid from the dorsal cortical of the ulna having its most prominent point as a reference is paramount for orthopedic surgeons. This allows meticulous planning and execution of the procedure, ensuring proper placement of K-wires or screws. This precision minimizes the risk of complications, such as nerve or vessel injury, and decreases the likelihood of implant malposition, ultimately optimizing surgical outcomes and enhancing patient recovery.

This study aimed to define a practical range for safely targeting the coronoid process from the dorsal cortex of the ulna in surgical practice. To achieve this goal, we conducted a retrospective anatomical study based on CTs. Our results showed that, using the most prominent point of the posterior cortex of the ulna as a reference, the coronoid process could be targeted within a range of 21.3 mm to 35.1 mm (30%-60% of the IED) in males and 19.3 mm to 31.9 mm (40%-60% of the IED) in females. A statistically significant difference was observed between the sexes, and thus, this gender difference must always be taken into account.

The anatomy of the elbow and coronoid process has been studied and extensively described in the literature.^13^ However, to the best of our knowledge, no one has previously described the location of the coronoid process or its position relative to the dorsal cortex of the ulna. Our study fills this gap in the literature by adding practicality and accessibility to the operating room workflow, saving time and improving patient safety and satisfaction.

Several approaches have been described for coronoid fracture fixation. In recent years, arthroscopy-assisted fixation has increasingly risen, being far less invasive and having experienced good outcomes. However, it may yield poor results if the operator lacks skill in arthroscopic surgery due to its long learning curve.^12^

Other studies have previously described the optimal placement of fixation devices on the coronoid process of the ulna from its posterior cortex.^7^ However, it is the first study of its kind to describe a safe interval for targeting the coronoid process from the posterior cortex of the ulna in a practical way.

However, it's important to acknowledge certain limitations of this study. This study did not account for either the mediolateral deviation or the approach angle of the Kirschner wire, both of which could affect accurate engagement with the coronoid. Additionally, a wire that is too small may be prone to slight bending and trajectory deviations. Further studies are needed to investigate these aspects. Although accurate and collected using a defined standard methodology, the analyses were based on radiological imaging, so the measurements obtained may differ slightly from actual anatomical structures. Further in vivo studies are needed to validate our findings. Additionally, a limitation of our study is that we did not consider the Proximal Ulna Dorsal Angulation in our measurement method. Nevertheless, this should not pose a significant issue, as the error introduced is likely to be systematic and therefore predictable.^19^ Further, more complex and comprehensive studies, are needed to investigate these points in greater detail.

Future research in this area could explore the application of these findings first on cadavers and subsequently in a prospective clinical setting with a larger and more diverse patient population. In addition, studying the feasibility of integrating APEX, BASE, IED, APEX/IED and BASE/IED measurements into preoperative planning software or intraoperative navigation systems could provide real-time decision support for the surgeon.

## Conclusion

This study contributes to establishing a practical range for the safe targeting of the coronoid process from the dorsal cortex of the ulna in surgical practice. By applying these concepts in clinical practice, orthopedic surgeons can achieve greater precision, minimize the risk of complications and malpositioning, and improve clinical outcomes. Maintaining a perpendicular trajectory to the dorsal cortex of the olecranon, within a safe range of 2-3.5 cm from its most prominent point, may allow for a secure approach to the coronoid. While further research is warranted to validate and improve these findings, as well as to explore their clinical implications, this study underscores the importance of anatomical considerations in orthopedic surgery and highlights avenues for future investigation.

## Disclaimers

Funding: No funding was disclosed by the authors.

Conflicts of interest: The authors, their immediate families, and any research foundations with which they are affiliated have not received any financial payments or other benefits from any commercial entity related to the subject of this article.
